# Evaluating the General Population of Saudi Arabia for Their Knowledge, Attitudes, and Practices Towards Dementia

**DOI:** 10.7759/cureus.49865

**Published:** 2023-12-03

**Authors:** Rofayda M Mohamad, Meriam Saleh A Alsaeed, Abdulrahman H Merdad, Deemah M Alghaith, Faisal M Binnshwan, Renad A Albusaad, Maryam Y Almuslem, Razan A Alamri, Hosam Hadi Hussan Awaji, Maisa N Alqahtani, Anfal A Alamrani

**Affiliations:** 1 Department of Preventive Medicine, King Salman Armed Forces Hospital in Northwestern Region, Tabuk, SAU; 2 College of Medicine, King Faisal University, Al-Ahsa, SAU; 3 College of Medicine, King Saud Bin Abdulaziz University for Health Sciences, Jeddah, SAU; 4 College of Medicine, King Saud Bin Abdulaziz University for Health Sciences, Riyadh, SAU; 5 College of Medicine, King Saud University, Riyadh, SAU; 6 Neurology, King Faisal University, Al Hofuf, SAU; 7 College of Medicine, Taif University, Taif, SAU; 8 College of Medicine, King Abdulaziz University, Jeddah, SAU; 9 College of Medicine, King Khalid University, Abha, SAU; 10 College of Medicine, University of Tabuk, Tabuk, SAU

**Keywords:** saudi arabia, practice, attitude, knowledge, dementia

## Abstract

Introduction

Dementia, a prevalent neurological condition, has a significant global impact on individuals and communities. Despite affecting approximately 50 million people worldwide, with an expected tripling by 2050, there are currently no widely available disease-modifying treatments. Recent efforts have concentrated on strategies involving legislation, regulations, and population-wide initiatives to address dementia risk, diagnosis, and care.

Methods

This cross-sectional survey engaged 6123 participants in Saudi Arabia, utilizing a multistage sampling design across provinces and cities. The study aimed to investigate the knowledge, attitudes, and practices of the Saudi Arabian general population regarding dementia.

Results

Participants displayed diverse opinions on dementia knowledge, with females exhibiting higher rates of knowledge, attitudes, and practices than males. Notably, 97.2% of females were aware of dementia compared to 78% of males. The perception of dementia as a healthcare priority was significantly higher in females (84.1%) than in males (59.6%). Older females (≥65) were identified as the age group most associated with dementia (92.50%) compared to males (71.10%).

Conclusion

While participants demonstrated excellent knowledge of hearing about dementia, understanding symptoms, and identifying modifiable factors, their knowledge regarding prevention and curability was found to be inadequate. A significant gender association was observed, with females exhibiting higher knowledge, attitudes, and practices than males.

## Introduction

Dementia stands out as a major public health concern associated with the aging demographic, characterized by the gradual loss of memory, cognitive function, or both, hindering daily activities while consciousness is retained. Alzheimer's disease is widely recognized as the predominant global cause of dementia, with other contributors such as frontotemporal dementia, vascular disorders, and dementia with Lewy bodies [1]. The profound impact of dementia extends beyond cognitive aspects, significantly influencing social behavior and complicating the lives of both those affected and their caregivers. It is noteworthy that dementia holds the sixth position among the leading causes of death worldwide [2].

Dementia encompasses a cluster of brain disorders affecting behavior, memory, and cognition, resulting in significant morbidity and dependence among the elderly. The global prevalence of dementia exceeds 55 million seniors, a number projected to soar to 131.5 million by 2050 [3]. Mild cognitive impairment (MCI) serves as a transitional stage between typical age-related cognitive decline and full-fledged dementia, marked by an estimated annual conversion rate of 20% [4]. To impede the advancement of dementia, cognitive training programs and the modification of risk factors stand out as viable strategies [5].

The aging population of Saudi Arabia is on the rise. In 2016, the segment of the population aged over 60 constituted 6.5%, totaling 1.3 million, and this figure is projected to climb to around 10 million, constituting 25% of the total population, by 2050 [6]. Moreover, the nation's life expectancy is expected to elevate from 74 to 82 years. As longevity increases, the likelihood of cognitive impairment is anticipated to surge. Additionally, the global prevalence of dementia is forecasted to escalate over the next two decades [7].

Recent data from the World Health Organization (WHO) indicates that the global prevalence of dementia is approximately 55 million, with an annual increase in the incidence of nearly 10 million people receiving a diagnosis. Additionally, a study conducted in Saudi Arabia reported that dementia affected 16.6% of senior patients [1].

Certain Western countries have developed diverse models of dementia care for primary care, often termed "collaborative care" or "patient-centered care," aimed at addressing the complex healthcare needs of patients and caregivers and facilitating interdisciplinary treatment [8]. Dementia care in a primary care setting has proven beneficial for both patients and their caregivers [9]. General practitioners (GPs) have demonstrated positive attitudes toward diagnosing and caring for dementia patients [10]. However, persistent challenges exist. For instance, GPs may lack confidence in making early diagnoses or screening patients before referring them to specialists [11]. Recommendations have been made for enhanced education for GPs to overcome these challenges [12].

Numerous research findings suggest that there is potential for improving diverse populations' attitudes, knowledge, and literacy about dementia [11]. To fill in these gaps and provide correct information on dementia risk reduction techniques, awareness campaigns and educational interventions should be created. In general, it is critical to raise the public, medical professional, and community health workers understanding and awareness of dementia-related issues. The aim of this study is to assess the effectiveness of awareness campaigns and educational interventions in enhancing attitudes, knowledge, and literacy about dementia risk reduction techniques across diverse populations. It is possible to lessen the effects of dementia and enhance the quality of life for both those who are caring for someone with dementia and those who are not by encouraging early detection, risk reduction, and appropriate care practices.

## Materials and methods

Study design

The research employed a cross-sectional study design, characterized by observational data collection at a specific time point. Utilizing a multistage sampling design, participants were chosen from various provinces, cities, and clusters within Saudi Arabia.

Study area

The research was conducted in Saudi Arabia, encompassing multiple provinces and cities throughout the country. With a diverse population and geographical distribution, the Kingdom of Saudi Arabia is divided into 13 provinces, including Riyadh, Qasim, Dammam, Khafji, Alhasa, Mecca, Medina, Jeddah, Asir, Najran, Jizan, Tabuk, and Jouf. This extensive coverage provided a comprehensive setting to evaluate the knowledge, attitudes, and practices of the general population regarding dementia.

Study setting

The data collection for this study was conducted in various settings within Saudi Arabia, encompassing both urban and rural areas. Community centers, public spaces, educational institutions (e.g., universities, colleges), and residential areas were included as study settings. The study duration was six months, spanning from May to November 2023.

Study population

The study encompassed individuals aged 18 years and above who resided in Saudi Arabia. The investigation targeted the general population, ensuring representation from diverse socioeconomic backgrounds, educational levels, occupations, and regions across the country.

Inclusion and exclusion criteria

Individuals aged 18 years and older were considered for inclusion, while those with a confirmed history of dementia or cognitive impairment were excluded from the study.

Sample size

The sample size for this study was calculated using the following formula:

$$n = \frac{Z^{2}*p*q}{d^{2}}$$

Where n is the required sample size, Z is the confidence level (standard value of 1.96 for 95% confidence), p is the estimated proportion of the population with adequate knowledge about pancreatitis (based on previous studies), q=1−p, and d is the margin of error (5% or 0.05). Assuming an estimated proportion of 50% and a margin of error of 5%, the minimum sample size was approximately 380 participants.

Sampling technique

A multi-stage sampling approach was employed, involving the selection of provinces, cities, and clusters within Saudi Arabia. In the first stage, random sampling was used to select a specific number of provinces from the sampling frame, ensuring geographic representation and diversity across Saudi Arabia. In the second stage, random sampling was employed to select a predetermined number of cities within each chosen province, representing various urban and rural areas. In the third stage, within each selected city, clusters were formed based on predefined criteria, such as neighborhoods or specific areas. Random sampling was then used to select a suitable number of clusters within each city. The selection process aimed to include a reasonable number of participants from different provinces of Saudi Arabia to ensure diversity and representation in the study. This technique ensured that the sample was representative of the study population and provided a sufficient number of participants for the study.

Data collection tools

The data were collected by multiple data collectors using a structured interviewing technique with a questionnaire. The survey was modified from one previously published [13]. The questionnaire compiled for the study consisted of 27 questions divided into three groups: the first five questions focused on the participants' demographics, the following eight questions assessed knowledge, and the last 14 questions examined attitude and practice. All data collectors were trained in the Arabization of the questions through a workshop designed for this purpose to ensure consistency in data collection. A pilot study was conducted among 25 participants not included in the main study to assess the clarity, understandability, relevance, and validity of the questionnaire items.

Data analysis plan

The data were analyzed using SPSS software, version 28 (IBM Corp., Armonk, NY). Descriptive statistics were employed to summarize the data. The Chi-square test was utilized to examine associations between variables. A p-value less than 0.05 was considered statistically significant.

Ethical consideration

The Institutional Review Board (IRB) of King Salman Armed Forces Hospital granted ethical clearance for this study (Approval Number: KSAFH-REC-2023-524). Prior to their participation in the trial, all participants provided verbal informed consent. Stringent measures were implemented to uphold the confidentiality and privacy of each participant.

## Results

The demographic information revealed that the total number of participants was 6123, with Saudi participants comprising the majority (n=5807, 94.8%). The female frequency (n=4270, 69.7%) exceeded that of males (n=1853, 30.3%). The majority of the study population fell within the age range of 21-30 years old (n=2431, 39.7%). Regarding education, the majority of participants had a university-level education (n=7303, 70.3%), and the predominant occupation level was students (n=2896, 47.3%), in Table 1.

**Table 1:** Sociodemographic characteristics of the study participants (n= 6123) n: Number of participants; %: Percentage of participants.

Variable	Classification	N	%
Nationality	Saudi	5807	94.8
	Non- Saudi	316	5.2
Gender	Female	4270	69.7
	Male	1853	30.3
Age groups (years)	Less than 20 years	1123	18.3
	21-30 years	2431	39.7
	31-40 years	474	7.7
	41-50 years	951	15.5
	51-60 years	876	14.3
	>60 years	268	4.4
Education Level	Elementary school	70	1.1
	High school	1172	19.1
	Higher education	435	7.1
	Middle school	99	1.6
	No education	44	0.7
	University	4303	70.3
Occupation	Employed	1781	29.1
	Homemaker	408	6.7
	Retired	809	13.2
	Student	2896	47.3
	Unemployed	229	3.7

As illustrated in Table 2, the general responses of the study participants indicated an insufficient level of knowledge about dementia. Most participants correctly identified the age ≥ 65 years as when dementia could affect (n=5266, 86%). However, the understanding that dementia is a normal part of aging was inadequate, with (n=2723, 44.5%) in agreement and (n=2462, 40.2%) in disagreement. Inadequate responses were observed regarding the hereditary nature of dementia, where (n=1909, 31.2%) answered yes, and (n=2620, 42.8%) answered no. A significant portion of participants prioritized dementia as a healthcare concern (n=4698, 76.7%). Short-term memory loss was correctly identified as the most common symptom by the majority (n=4318, 70.52%). However, there was an inadequate understanding of whether dementia can be prevented by modifying certain factors, with the highest rate (n=2635, 43.03%) believing dementia cannot be prevented. Additionally, (n=2815, 46%) were uncertain about whether dementia could be cured with medication. Those who reported not knowing anyone with dementia were higher (n=2477, 56%). Participants expressed concern about developing dementia themselves when they become older, representing a significant proportion (n=3434, 56.1%). Responses regarding hiding the dementia diagnosis of a relative were inadequate, with (n=3373, 55.15%) indicating "not applicable." Regarding the health impact of caring for a relative with dementia, (n=3568, 58.3%) answered "not applicable." A substantial number of participants (n=2927, 47.8%) did not know whether doctors and nurses ignore people with dementia, while (n=2259, 36.9%) answered no. The majority expressed no shame about having a relative with dementia (n=3830, 62.6%) and were worried about close family members or friends developing dementia (n=3521, 57.5%). Most participants agreed to visit doctors if they experienced memory problems (n=4579, 74.8%) and would encourage others to do the same (n=5114, 83.5%). The majority believed they could know the diagnosis if they suffered from dementia (n=4425, 72.3%). Regarding informing patients about their dementia diagnosis when caring for someone, 2886 (47.1%) participants agreed. However, the majority did not perceive a dementia diagnosis as a death sentence (n=3481, 56.9%). About whether dementia is worse for family and friends than for the person with dementia, the majority answered yes (n=3514, 57.4%). Regarding the ability of people with dementia to drive, the majority answered no (n=4842, 79.1%).

**Table 2:** Knowledge, attitudes, and practices toward dementia disease n: Number of participants; %: Percentage of participants.

Question	Response	N	%
Have you heard the word “dementia”?	Yes	5595	91.4
	No	528	8.6
Which age group does dementia affect?	Adults > 18 years	36	0.6
	Any age	256	4.2
	Children ≤ 12 years	23	0.4
	Don’t know	507	8.3
	Old age group ≥ 65 years	5266	86
	Teenagers 12-18 years	35	0.6
Do you think dementia is a normal part of aging?	Don’t know	938	15.3
	No	2462	40.2
	Yes	2723	44.5
Is dementia hereditary (family linked, i.e., if your parents have/had dementia then you shall get it too)	Don’t know	1594	26
	No	2620	42.8
	Yes	1909	31.2
Is dementia a healthcare priority?	Don’t know	960	15.7
	No	465	7.6
	Yes	4698	76.7
Which of the following symptoms may a person with dementia experience?	Impaired judgment	3236	52.85
	Difficulty communicating	3645	59.53
	Problems learning new skills	3158	51.58
	Short-term memory loss	4318	70.52
	Altered sleep pattern	1691	27.62
	Inability to carry out familiar tasks	3209	52.41
	Hearing loss	849	13.87
	Personality changes	3077	50.25
	Loss of appetite	1243	20.30
	Depression	2311	37.74
	Hallucination	2135	34.87
	Delusions	2586	42.23
	Loss of ability to generate new ideas	2250	36.75
	Change in behavior	2046	33.41
	Long-term memory problem	2931	47.87
	Weakness of both limbs	896	14.63
	Loss of vision	331	5.41
	Don’t know	592	9.67
Do you think dementia can be prevented by modifying the factors given below?	Cholesterol levels	1181	19.29
	Healthy diet	2088	34.10
	Depression	1706	27.86
	Dementia cannot be prevented	2635	43.03
	Diabetes	2016	32.93
	Heart diseases	873	14.26
	Increased blood pressure	1444	23.58
	Obesity	1269	20.73
	Smoking	1660	27.11
	Excessive alcohol intake	2318	37.86
Do you think people with dementia can be cured with medication?	Yes	1508	24.6
	No	1800	29.4
	Don’t know	2815	46
Do you know anyone or have you known anyone with dementia?	Yes	2646	43.2
	No	2477	56.8
As you get older do you worry about developing dementia?	Yes	3434	56.1
	No	1520	24.8
	Don’t know	1169	19.1
As a carer or family member of the patient, have you hidden the diagnosis of dementia from people?	Yes	333	5.4
	No	2417	39.5
	Not applicable	3373	55.1
Has your health suffered as a result of the caring responsibilities of a relative with dementia?	Yes	744	12.2
	No	1811	29.6
	Not applicable	3568	58.3
Do you think doctors and nurses ignore people with dementia?	Yes	937	15.3
	No	2259	36.9
	Don’t know	2927	47.8
Are you ashamed of having a relative with dementia?	Yes	210	3.4
	No	3830	62.6
	Not applicable	2083	34
You worry about close family members or close friends developing dementia.	Yes	3521	57.5
	No	1503	24.5
	Don’t know	1099	17.9
If you were experiencing memory problems, would you see a doctor?	Yes	4579	74.8
	No	513	8.4
	Don’t know	1031	16.8
If someone close to you was experiencing a memory problem, would you encourage them to see a doctor?	Yes	5114	83.5
	No	297	4.9
	Don’t know	712	11.6
If you were suffering from dementia, you would like to be told your diagnosis?	Yes	4425	72.3
	No	692	11.3
	Don’t know	1006	16.4
If someone you were caring for was suffering from dementia, would you want them to be told the diagnosis?	Yes	2886	47.1
	No	2039	33.3
	Don’t know	1198	19.6
Is the diagnosis of dementia like a death sentence?	Yes	1244	20.3
	No	3481	56.9
	Don’t know	1398	22.8
Is it worse for family and friends than for the person with dementia?	Yes	3514	57.4
	No	936	15.3
	Don’t know	1673	27.3
Do you think people with dementia should be allowed to drive?	Yes	310	5.1
	No	4842	79.1
	Don’t know	971	15.9

The values mentioned in Table 3 revealed a highly significant association among all participants in terms of knowledge, attitude, and practice of dementia, except for the question "Do you know anyone or have you known anyone with dementia?" where the association was non-significant (p=0.461). In terms of hearing about dementia, females exhibited higher knowledge (n=4149, 97.2%) compared to males (n=1446, 78%). Similarly, awareness of the age affected by dementia was more prevalent in females (n=3948, 92.50%) than in males (n=1318, 71.10%). Regarding the perception of dementia as a normal part of aging, females showed a higher percentage (n=2040, 47.8%) than males (n=683, 36.9%). Disagreement about the hereditary nature of dementia was more common in females (n=1906, 44.6%) than in males (n=71438, 5%). Concerning the prioritization of dementia as a healthcare issue, females demonstrated higher agreement (n=3593, 84.1%) compared to males (n=1105, 59.6%). Short-term memory loss was identified as the most common symptom by both genders, with higher frequencies in females (n=3060, 49.98%) compared to males (n=1258, 20.55%). Responses regarding the prevention of dementia through modifying factors showed that the highest frequency of answers in females was "Dementia cannot be prevented" (n=1917, 31.31%), while in males, it was "Excessive alcohol intake" (n=789, 12.89%). Both males (n=953, 51.4%) and females (n=1862, 43.6%) expressed uncertainty about whether dementia could be cured. Participants from both genders reported not knowing anyone with dementia, with females at (n=2427, 56.8%) and males at (n=1050, 56.7%). Regarding hiding the diagnosis of dementia, females responded "Not applicable" more frequently (n=2443, 57.2%) than males (n=930, 50.2%). Responses about the health impact of caring for a relative with dementia showed that both males and females marked "Not applicable" as the predominant answer (n=2613, 61.2%) for females and (n=955, 51.55%) for males. The perception that doctors and nurses ignore people with dementia was commonly expressed as "Don't know" by both females (n=1931, 45.2%) and males (n=996, 53.8%). Both genders reported no shame about having a relative with dementia, with females at (n=2700, 63.2%) and males at (n=1130, 60%). Worry about close family members or friends developing dementia was prevalent in both females (n=2680, 62.8%,) and males (n=841, 45.4%). Positive responses about visiting doctors if experiencing memory problems were higher in females (n=3429, 80.3%) than in males (n=1150, 62.1%). Encouraging closer persons to visit doctors if they suffered from memory problems was more common in females (n=3819, 89.4%) than in males (n=1295, 69.9%). Responses about informing a patient about their dementia diagnosis when caring for someone showed a higher frequency of "Yes" in females (n=2089, 48.9%) and males (n=797, 43%). Regarding whether the diagnosis of dementia is perceived as a death sentence, the negative response was the highest frequency for both females (n=2553, 59.8%) and males (n=928, 50.15%). In response to whether it is worse for family and friends than for the person with dementia, the highest frequency of "Yes" was observed in females (n=2574, 60.3%,) and males (n=940, 50.7%). Both genders were in agreement about not allowing people with dementia to drive, with females at (n=3608, 84.5%) and males at (n=1234, 66.6%).

**Table 3:** Association between gender groups and dementia knowledge, attitudes, and practices *Fisher’s Exact Test A p-value of ≤0.05 and ≤0.01 was considered the cut-off point for statistically significant differences between variables.

Variables	Responses of the participants	Females	Males	p-value
		4270 (69.7%)	1853 (30.3%)	
Have you heard the word “dementia”?	Yes	4149 (97.20)	1446 (78.00)	0.0001*
	No	121 (2.80)	407 (22.00)	
Which age group does dementia affect?	Old age group ≥ 65 years	3948 (92.50)	1318 (71.10)	0.0001
	Adults > 18 years	12 (0.30)	24 (1.30)	
	Teenagers 12-18 years	23 (0.50)	12 (0.60)	
	Children ≤ 12 years	11 (0.30)	12 (0.60)	
	Any age	141 (3.30)	115 (6.20)	
	Don’t know	135 (3.20)	372 (20.10)	
Do you think dementia is a normal part of aging?	Yes	2040 (47.80)	683 (36.90)	0.0001
	No	1803 (42.20)	659 (35.60)	
	Don’t know	427 (10.00)	511 (27.60)	
Is dementia hereditary (family linked, i.e., if your parents have/had dementia then you shall get it too)	Yes	1488 (34.80)	421 (22.70)	0.0001
	No	1906 (44.60)	714 (38.50)	
	Don’t know	876 (20.50)	718 (38.70)	
Is dementia a healthcare priority?	Yes	3593 (84.10)	1105 (59.60)	0.0001
	No	259 (6.10)	206 (11.10)	
	Don’t know	418 (9.80)	542 (29.20)	
Which of the following symptoms may a person with dementia experience?	Impaired judgment	2241 (36.60)	995 (16.25)	0.0001
	Difficulty communicating	2586 (42.23)	1059 (17.30)	
	Problems learning new skills	2252 (36.78)	906 (14.80)	
	Short-term memory loss	3060 (49.98)	1258 (20.55)	
	Altered sleep pattern	1266 (20.68)	436 (7.12)	
	Inability to carry out familiar tasks	2298 (37.53)	911 (14.88)	
	Hearing loss	652 (10.65)	197 (3.22)	
	Personality changes	2267 (37.02)	813 (13.28)	
	Loss of appetite	936 (15.29)	307 (5.01)	
	Depression	1726 (28.19)	585 (9.55)	
	Hallucination	1594 (26.03)	541 (8.84)	
	Delusions	1893 (30.92)	693 (11.32)	
	Loss of ability to generate new ideas	1570 (25.64)	668 (10.91)	
	Change in behavior	1498 (24.47)	548 (8.95)	
	Long-term memory problem	2026 (33.09)	895 (14.62)	
	Weakness in both limbs	580 (9.47)	271 (4.43)	
	Loss of vision	267 (4.36)	64 (1.05)	
	Don’t know	356 (5.81)	236 (3.85)	
Do you think dementia can be prevented by modifying the factors given below?	Excessive alcohol intake	1529 (24.97)	789 (12.89)	0.0001
	Diabetes	862 (14.08)	593 (9.68)	
	Obesity	785 (12.82)	484 (7.90)	
	Smoking	1017 (16.61)	574 (9.37)	
	Increased blood pressure	998 (16.30)	446 (7.28)	
	Cholesterol levels	820 (13.39)	361 (5.90)	
	Healthy diet	1486 (24.27)	590 (9.64)	
	Depression	1171 (19.12)	535 (8.74)	
	Heart diseases	617 (10.08)	256 (4.18)	
	Dementia cannot be prevented	1917 (31.31)	718 (11.73)	
Do you think people with dementia can be cured with medication?	Yes	1101 (25.80)	407 (22.00)	0.0001
	No	1307 (30.60)	493 (26.60)	
	Don’t know	1862 (43.60)	953 (51.40)	
Do you know anyone or have you known anyone with dementia?	Yes	1843 (43.20)	803 (43.30)	0.461
	No	2427 (56.80)	1050 (56.70)	
As you get older do you worry about developing dementia?	Yes	2663 (62.40)	771 (41.60)	0.0001
	No	1027 (24.10)	493 (26.60)	
	Don’t know	580 (13.60)	589 (31.80)	
As a carer or family member of the patient, have you hidden the diagnosis of dementia from people?	Yes	196 (4.60)	137 (7.40)	0.0001
	No	1631 (38.20)	786 (42.40)	
	Not applicable	2443 (57.20)	930 (50.20)	
Has your health suffered as a result of the caring responsibilities of a relative with dementia?	Yes	539 (12.60)	205 (11.10)	0.0001
	No	1118 (26.20)	693 (37.40)	
	Not applicable	2613 (61.20)	955 (51.50)	
Do you think doctors and nurses ignore people with dementia?	Yes	646 (15.10)	291 (15.70)	0.0001
	No	1693 (39.60)	566 (30.50)	
	Don’t know	1931 (45.20)	996 (53.80)	
Are you ashamed of having a relative with dementia?	Yes	123 (2.90)	87 (4.70)	0.001
	No	2700 (63.20)	1130 (60.00)	
	Not applicable	1447 (33.90)	636 (34.30)	
You worry about close family members or close friends developing dementia.	Yes	2680 (62.80)	841 (45.40)	0.0001
	No	1039 (24.30)	464 (25.00)	
	Don’t know	551 (12.90)	548 (29.60)	
If you were experiencing memory problems, would you see a doctor?	Yes	3429 (80.30)	1150 (62.10)	0.0001
	No	305 (7.10)	208 (11.20)	
	Don’t know	536 (12.60)	495 (26.70)	
If someone close to you was experiencing a memory problem, would you encourage them to see a doctor?	Yes	3819 (89.40)	1295 (69.90)	0.0001
	No	183 (4.30)	114 (6.20)	
	Don’t know	268 (6.30)	444 (24.00)	
If you were suffering from dementia, would you like to be told your diagnosis?	Yes	3312 (77.60)	1113 (60.10)	0.0001
	No	474 (11.10)	218 (11.80)	
	Don’t know	484 (11.30)	522 (28.20)	
If someone you were caring for was suffering from dementia, would you want them to be told the diagnosis?	Yes	2089 (48.90)	797 (43.00)	0.0001
	No	1577 (36.90)	462 (24.90)	
	Don’t know	604 (14.10)	594 (32.10)	
Is the diagnosis of dementia like a death sentence?	Yes	925 (21.70)	319 (17.20)	0.0001
	No	2553 (59.80)	928 (50.10)	
	Don’t know	792 (18.50)	606 (32.70)	
Is it worse for family and friends than for the person with dementia?	Yes	2574 (60.30)	940 (50.70)	0.0001
	No	664 (15.60)	272 (14.70)	
	Don’t know	1032 (24.20)	641 (34.60)	
Do you think people with dementia should be allowed to drive?	Yes	196 (4.60)	114 (6.20)	0.0001
	No	3608 (84.50)	1234 (66.60)	
	Don’t know	466 (10.90)	505 (27.30)	

## Discussion

Globally, dementia has emerged as a significant public health concern, affecting 50 million individuals at present, with projections indicating a surge to 152 million by 2050 as the global population ages [14]. This trend is not exclusive to specific regions, as the prevalence of dementia in the Netherlands is expected to rise from 280,000 in 2018 to over 620,000 by 2050 [15]. Saudi Arabia follows a similar trajectory, with an estimated dementia prevalence of 12.9%, surpassing previous local hospital-based studies and aligning with findings from Canada, Italy, and the Netherlands. Despite this, research on dementia in the Gulf region is relatively limited compared to the Western world [16]. Recent years have witnessed substantial progress in scientific knowledge pertaining to cognition and dementia. Advances in understanding the causes and risk factors, including genetic considerations, have been notable [17]. This growing body of knowledge underscores the importance of addressing dementia on a global scale and emphasizes the need for expanded research efforts, particularly in regions like the Gulf, where insights into this condition are relatively limited.

Our study encompassed 6,123 participants, spanning diverse age groups from under 20 years old to above 60 years old. Among the participants, (n=4,270, 69.7%,) were females, while males accounted for (n=1,853, 30.3%) participants. The majority of participants were Saudis, totaling (n=5,807, 94.8%), with non-Saudis comprising a smaller proportion at (n=316, 5.2%).

The majority of participants in our study demonstrated a heightened awareness of dementia, particularly in terms of having heard about it (n=5,595, 91.4%). This awareness surpassed the findings of a Chinese study, which reported a knowledge rate of (n=645, 65%) [18], and exceeded the results of the Aldharman et al. study, where the knowledge percentage was (n=1,300, 80.6%) [1]. Moreover, a substantial portion of our participants (n=5,266, 86%) correctly identified the age group most susceptible to dementia as the elderly, specifically those aged ≥ 65 years. These results align with those reported by Chan et al. [19].

In contrast to a previous survey that asserted "dementias are not an inevitable part of aging," a higher proportion of participants (n=2,723, 44.5%) in our study believed that dementia is a typical part of the aging process [20]. Participants' responses to the question "Is dementia inherited?" indicated inadequacy, with (n=2,620, 42.8%) responding "no," and (n=1,909, 31.2%) responding "yes." These findings contradicted a study by Paulson and Igo, which emphasized that "genetic factors are now recognized to play an important role in most age-related dementias" [21], and another study that reported "25% of all people aged 55 and older have a family history of dementia" [22].

The majority of the respondents recognized dementia as a healthcare priority (n=4,698, 76.7%), aligning with the global perspective presented by WHO [23] and the study by Frankish and Horton [24], which emphasized that "Today, nearly 50 million people worldwide have dementia, with this figure projected to increase to 75 million by 2030 and to 132 million by 2050." Regarding the main symptoms of dementia, the majority of participants identified short-term memory loss as the primary symptom (n=4,318, 70.5%), while long-term memory loss was also acknowledged (n=2,931, 47.87%). A study conducted in Jeddah, Saudi Arabia, supported our findings, reporting that memory loss represented (n=1,397, 82.3%) of respondents' answers [25].

The most frequently reported response by participants regarding the prevention of dementia was that "Dementia cannot be prevented" (n=2,635, 43.03%). These findings are consistent with those of Cations et al. [26], who observed that "nearly half of respondents agreed that dementia is a normal and non-preventable part of aging" based on systematic reviews and meta-analyses conducted between 2012 and 2017. Similar results were reported by Algahtani et al. [25], indicating that (n=509, 30%) of participants believed Alzheimer's disease could be treated with medications, and (n=418, 24.6%) believed there was no treatment. Approximately (n=1,508, 24.6%) of participants believed dementia could be treated with medications, (n=1,800, 29.4%) believed there is no treatment, and (n=2,815, 46%) were uncertain.

All participants exhibited a highly significant association between gender groups and their knowledge, attitude, and practices related to dementia (p=0.0001), except for the question "Do you know anyone or have you known anyone with dementia?" where the association was non-significant (p=0.461).

The comprehensive knowledge outcomes indicated that females exhibited superior knowledge, attitudes, and practices concerning dementia compared to males. These results differ from the findings of Badawoud et al. [27], who reported a non-significant gender-based difference in dementia knowledge (p=0.25). However, our results align with Guo et al. [28], who found a significant divergence in knowledge and attitude toward dementia between females and males (p=0.009). Specifically, females demonstrated a positive attitude (n=653, 75.8%), whereas males exhibited a positive attitude (n=209, 24.2%).

Limitations of the study

Comparable to a cross-sectional design, this approach imposes limitations on inferring potential causal relationships due to the collection of data occurring only once over a brief period. There is a possibility of social desirability bias affecting some participant responses, cautioning against definitive interpretations of attitudes. Nevertheless, the study boasts strengths such as the diverse representation of provinces among participants and substantial sample size, contributing to a commendable level of result representativeness and generalizability.

## Conclusions

To the best of our knowledge, this study is the first to comprehensively address dementia knowledge, attitudes, and practices across various provinces and cities in Saudi Arabia. The results reveal a mixed perception among the general population in Saudi Arabia concerning dementia. While overall knowledge about hearing of dementia, recognizing dementia as a healthcare priority, and identifying the age at which dementia can manifest was commendable, there were gaps in understanding certain aspects. Notably, participants exhibited a strong consensus that short-term memory loss was the most prevalent symptom of dementia. The study found a highly significant association between gender and dementia knowledge, attitudes, and practices, with the exception of familiarity with someone who has dementia. Importantly, our findings indicate that females displayed a higher association with dementia knowledge, attitudes, and practices compared to males.
